# How food insecurity affects children’s behavior problems in early childhood: The nutrition and family stress pathways

**DOI:** 10.1371/journal.pone.0294109

**Published:** 2024-01-03

**Authors:** Xuejiao Chen, Wei-Jun Jean Yeung

**Affiliations:** 1 Yong Loo Lin School of Medicine, National University of Singapore, Singapore, Singapore; 2 Agency for Science, Technology and Research (A*STAR), Singapore, Singapore; University of Agriculture Faisalabad, PAKISTAN

## Abstract

This study examines how household food insecurity shapes young children’s behavior problems in Singapore. The analysis is based on two waves of data collected before and during COVID-19 from a nationally representative sample of 2,601 children in the Singapore Longitudinal Early Development Study (SG-LEADS, M_age_ = 4.5 at wave 1, M_age_ = 6 at wave 2). Results based on propensity score matching, fixed effects analysis and lagged-variable models show a positive association between household food insecurity and children’s behavior problems both concurrently and over a two-year period. Two mediating pathways of this association are identified—children’s dietary intake and family stress. Children in food-insecure households tend to consume fewer vegetables and more sugar-sweetened beverages and carbohydrates, which is associated with elevated behavior problems. Parents in food-insecure households exhibit greater emotional distress, diminished parental warmth, and increased punitive parenting practices, also contributing to their children’s behavior problems. The family stress pathway has a stronger explanatory power than the nutrition pathway on children’s behavior problems. This study reveals that food insecurity is a risk factor for children’s behavior problems in early childhood which can lead to later developmental vulnerabilities for children in financially deprived families.

## Introduction

Food insecurity refers to the “limited or uncertain availability of nutritionally adequate and safe foods, and limited or uncertain ability to acquire acceptable foods in socially acceptable ways” [1]. Studies have revealed multi-faceted negative implications of food insecurity on early childhood development extending beyond physical health to socioemotional development. Early childhood is a crucial period characterized by fast growth, heightened susceptibility to environmental influence and great malleability and development in this critical stage have far-reaching implications for later development [2,3]. Setbacks in early childhood will likely persist into later life stages, contributing to adolescent delinquencies and mental health issues during adulthood [4–6]. This topic is particularly salient given the increase in food insecurity during the COVID-19 pandemic around the world [7,8]. To help inform policy interventions, it is imperative to understand the various mechanisms through which food insecurity affects children’s socioemotional development in early childhood.

Studies in North America show that household food insecurity is positively associated with children’s behavior problems [9–17]. For example, Whitaker (2006) find that American preschool children living in food-insecure households show more behavior problems than their food-secure counterparts net of controls [14]. Kimbro and Denney (2015) show that transitioning into food insecurity and the persistence of that status between kindergarten and first grade correlate with elevated internalizing and externalizing behavior problems in American children [9]. To explain this association, scholars have identified parents’ subjective wellbeing and parenting practices as mediators that explain the link between food insecurity and children’s behavior problems [18–20]. Nevertheless, several gaps in knowledge remain. First, the causal relationship between food insecurity and children’s behavior problems has not been firmly established as many studies use cross-sectional data or fail to account for the impact of time-invariant factors in the analysis. Second, the mechanism through which food insecurity impacts children’s social-emotional development is not well understood. Although family stress was examined in the current literature, less attention has been paid to other mediating mechanisms such as children’s nutrition, perhaps due to a lack of data.

This study aims to examine the causal relationship between food insecurity during the COVID-19 pandemic and young children’s behavior problems in a non-Western country and evaluate two mediating pathways of the association and their relative significance. Using longitudinal data collected before and during the COVID-19 from a nationally representative sample of households with preschool children in Singapore, we applied rigorous statistical methods including propensity score matching, fixed-effect models, and lagged-variable models to account for potential bias between food insecurity and children’s behavior. Moreover, we investigated the mediating pathways of food insecurity on children’s behavior from two distinct pathways—the nutrition pathway, which is rarely assessed in the current literature, and the family stress pathway. To our knowledge, it is among the first attempts to jointly examine the biological and social-emotional pathways that mediate the association between food insecurity and children’s behavior problems. This study will also provide new evidence in an Asian context where welfare assistance is generally not as generous as that in the Western world for international comparison.

## Review of the literature

### Impact of food insecurity on children’s behavior problems

Research demonstrates the adverse impact of food insecurity on children’s psychological functioning with children of food-insecure households exhibiting more behavior problems than their peers both in cross-sectional studies [14,15] and longitudinal studies [9–13,16,17]. In longitudinal studies, a 2-year study by Slopen on 4- to 16-year-old American children shows that persistent food insecurity increases children’s chance of showing internalizing behavior problems by 1.5 times and externalizing behavior problems by 2 times [12]. This study also reveals that transitioning into food insecurity leads to a 1.8 times higher chance of developing externalizing behavior problems during the follow-up period. Another longitudinal study conducted on Canadian children finds that those who experienced food insecurity during the first 4.5 years of life are 1.8 times more likely to exhibit symptoms of depression or anxiety and 3.1 times more likely to display hyperactivity or inattention between the ages of 4.5 and 8 [13]. Most of these studies face some methodological challenges. Studies with cross-sectional design (e.g., [14,15]) are weakened by the possibility of reverse causality, selection bias and unobserved confounders. In most longitudinal studies (e.g., [9,12,16]), researchers fail to account for unobserved time-invariant factors that can confound the association between household food insecurity and child development. One example of such time-invariant variables is prenatal conditions, including maternal health during pregnancy, which may lead to birth complications such as low birth weight, which in turn may affect children’s behavior problems [21]. Food insecurity is an extreme environmental stressor, which is closely related to other stressors such as family economic pressure, parental psychological health, single parenthood, marital discord and domestic violence [22,23]. While some studies have included some of the abovementioned risks as control variables, it remains a challenge to fully eliminate the selection bias that is not captured by the controlled factors. To establish the impact of food insecurity on child development, it is important to take such factors into account.

Only a handful of studies have examined the causal relationship between food insecurity and children’s behavioral outcomes [18,24,25]. These studies typically apply more robust analytical methods such as the fixed effects method and lagged models, using longitudinal datasets. Jyoti and colleagues [24] employ lagged models with two waves of Early Child Longitudinal Study-Kindergarten Cohort (ECLS-K 1999) to rule out the possibility of reverse causality. Huang and colleagues [18], focusing on a low-income sample in the Panel Study of Income Dynamics, and King [25], using the Fragile Families and Child Wellbeing Study, both employed fixed-effects models to account for the individual-level time-invariant factors. All these studies were conducted with American children. It is essential to conduct rigorous research in other socioeconomic contexts to improve the generalizability of existing findings and deepen our understanding of the impact of food insecurity in different regions of the world. This is particularly important as the COVID-19 pandemic has increased food insecurity significantly but with different impacts across countries. An objective of this paper is thus to provide rigorous empirical evidence of the impact of food insecurity during covid-19 pandemic on young children’s behavior in a non-Western country in Asia.

### Mediating mechanisms of food insecurity on behavior problems

Our second objective is to contribute to a nuanced understanding of the mediating mechanism through which food insecurity affects children. Research has been conducted to identify the mechanisms through which hunger may link to young children’s developmental outcomes. Childhood nutrition is a potential yet underexplored mechanism, probably due to a lack of detailed measures of children’s dietary intake [25]. Food insecurity can lead to undernutrition and micronutrient deficiencies, which in turn, directly and adversely affect children’s behavior problems [26,27]. Children in food-insecure households have limited access to nutritious meals, potentially leading to deficiencies in essential micronutrients like iron, zinc, and vitamins. These deficiencies have been linked to potential brain damage and impaired neuropsychological growth, especially in infants and toddlers [28–31]. For older children, food insecurity is associated with limited food choices. Food-insecure children tend to over-consume carbohydrate-rich foods which are typically more affordable and energy-dense due to their cost-effectiveness [32,33]. Moreover, several recent studies showed that food-insecure parents who feel stressed are more likely to use (unhealthy) food (e.g., sugar-sweetened food) as means of emotional regulation or reward to children [34,35]. These parents tend to de-emphasize the importance of a balanced meal and resort to pre-prepared food (e.g., fast food) for their children [34,36,37]. Consequently, food-insecure children’s diets may be disproportionately high in fat, refined sugar and sodium, but deficient in fruit, vegetable, and fiber [26,31,38]. High consumption of refined sugar and iron-deficiency anemia have been associated with hyperkinesia, inattention, and poor memory [13,27,39].

Another mechanism is through the primary caregiver’s mental well-being and parenting behavior [19,40,41]. It is well-established that family economic hardship may create emotional distress for parents leading them to use more punitive parenting behavior which is related to more behavior problems in children [42–44]. Food deprivation reflects a family’s struggle to afford basic necessities and difficulties in making ends meet. Managing these processes could be stressful and may create a sense of hopelessness and emotional distress for caregivers of children [45,46]. Parental distress can disrupt family functioning and cause family conflicts among family members and lead to less warmth and more harsh parenting practices [20,47,48]. Parental warmth and supportive parenting promote good adjustments in children while weak maternal responsiveness and frequent physical discipline correlate with elevated problem behavior among children [42,43]. Several studies indicate that parental characteristics, such as parental depressive affect, parenting aggravation, and parental warmth, mediate the effects of food insecurity on children’s externalizing behavior problems and internalizing behavior problems in the U.S. [16,20,40].

To the best of our knowledge, no study has examined the joint mediating roles of both children’s dietary intake and family stress in the relationship between food insecurity and children’s behavior problems. A few studies (for example, [34–36,49]) investigated how family stress affects parents’ feeding behavior and children’s eating habits within the context of food insecurity although they did not link these to children’s behavioral problems.

To fill the gaps in the extant literature mentioned above, the present study makes the following contributions: (1) We examined the causal impact of food insecurity on children’s behavior problems by employing robust statistical methods, including the propensity score matching, fixed-effects models and lagged-variable models with a recent nationally representative longitudinal dataset in Singapore, (2) We jointly tested two distinct mediating pathways: the nutrition pathway and the family stress pathway, and (3) The study adds new evidence on the relationship between food insecurity and child development in a highly developed, non-Western context where the public and private social support systems are different from the Western countries.

### Singapore context

Singapore is a wealthy city-state in Asia. It was ranked among the most food-secure nations in the world on the global food security index before the COVID-19 pandemic [50]. A recent study conducted in late 2019, however, reveals that around 10.4% of Singapore households suffer from insufficient food in the 12 months; among which 3.5% experience food insecurity at least once every month [51]. The incidence rose during the COVID-19 pandemic as the economic repercussions led to job and income losses for many parents [52–54]. This was not unique to Singapore, however, as evidenced by U.S. statistics indicating a doubling in food insecurity amidst the pandemic, with nearly one-third of households with children aged 5–17 facing food insecurity in 2020 [55].

In Singapore, the local transmission of the coronavirus was detected in January 2020. In response to the rising cases, a nationwide partial lockdown was enforced which included measures like social distancing, work-from-home for non-essential workers, and the closure of daycare and schools. Thereafter, different levels of restriction were in place from 2020 to 2022, adjusting based on the trend of community cases, significantly impacting the economy. This further worsened the food insecurity situation, with charities reporting increased demands for food support during the pandemic, including a new group that emerged due to the economic downturn [52,56,57]. However, to our knowledge, there has been scant study on how food insecurity during the pandemic in Asia has affected children’s development.

## Method

### Data and sample

The sample was drawn from two waves of the Singapore Longitudinal Early Development Study (SG-LEADS). The wave 1 (baseline) survey was conducted between 2018 and 2019 with a national sample of 5,005 Singaporean children aged below 7 in 3,477 Singapore households [58]. The study adopted a multi-stage probability sampling—clustered and stratified sampling strategy—with an oversample of the low-income population. Face-to-face in-home interviews with the primary caregiver (95% mother, 4% father) of the target child were conducted to collect a wide range of information about the household and the target child. In 2021, the children were followed up with 4,352 of them being successfully re-interviewed (a response rate of 87%). Written approval has been granted for the study by the National University of Singapore Institutional Review Board (reference No. S-17-326). Written informed consent was obtained from all participants for inclusion in the study.

The analytic sample of the present study includes 2,601 children who were 3 to 6 years old in the wave 1 study and participated in both waves (mostly 5 to 9 years old in the wave 2 study, 52% boys, 67% Chinese, see S1 Minimal dataset). Analysis of the sample attrition shows that primary caregivers with lower education, lower family income and live in rental flats were slightly more likely to drop out in wave 2. Sampling weights have been created to adjust for the initial selection probability and non-responses. A child-level weight was applied to the analysis.

### Analytical framework

**Fig 1:**
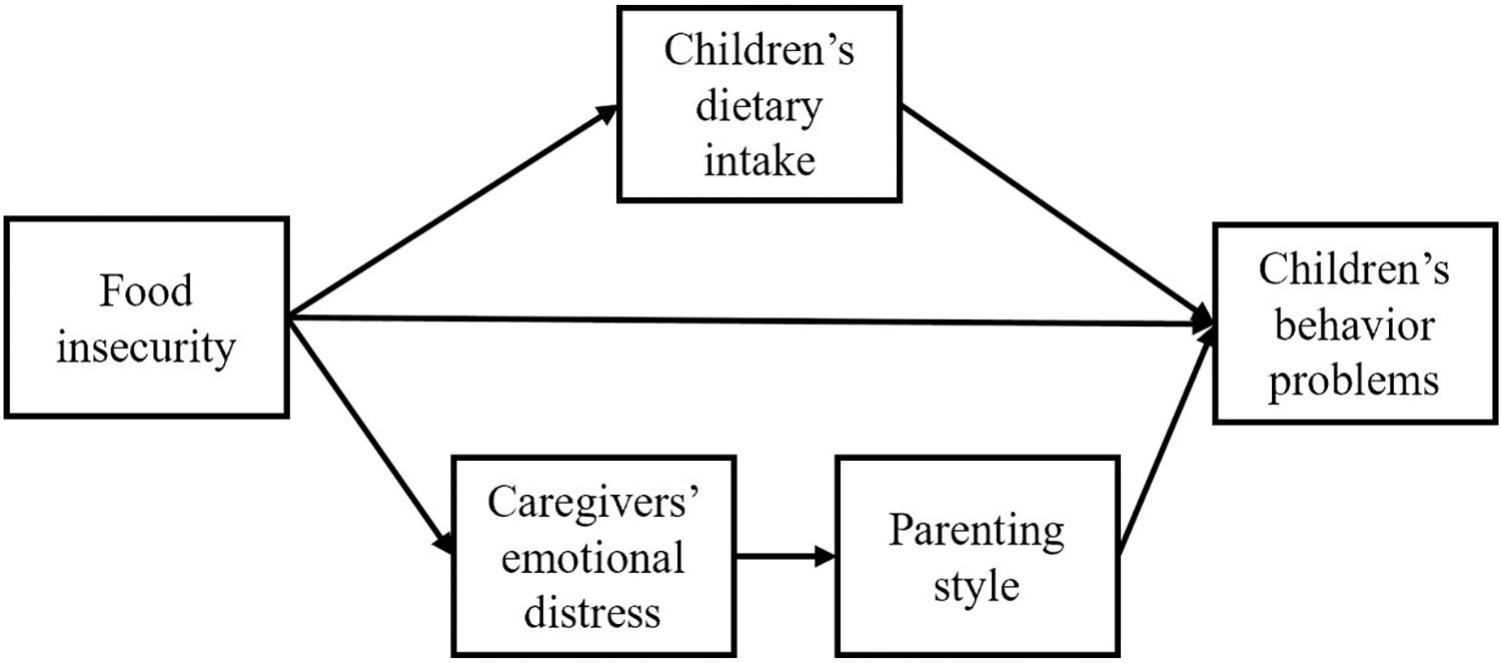
The conceptual framework.

Fig 1 depicts the conceptual framework that guides the analyses in this study. Food insecurity is viewed as directly affecting children’s dietary intake, which in turn contributes to increased children’s behavior problems. Food insecurity is also conceptualized to affect parental emotional distress, which affects parenting styles such as parental warmth and harsh discipline, further contributing to more behavior problems in children.

### Measures

#### Behavior problems

The dependent variable is children’s behavior problems index measured by the behavior problems scale [59]. The behavior problems consist of two subscales: **externalizing behavior problems and internalizing behavior problems** with the former including behavior such as aggression, delinquency, and hyperactivity, and the latter containing anxiety /depression and peer problems. Primary caregivers reported their children’s behavior in the past six months on a 3-point Likert Scale (1 = often true, 3 = not true). The items of each subscale were reversed coded and averaged to generate an externalizing behavior problems index (externalizing BPI, Cronbach’s α was 0.86) and an internalizing behavior problems index (internalizing BPI, Cronbach’s α was 0.88). The higher the score, the higher the child’s behavior problems.

**Food insecurity** was measured by three questions including whether the families ever worried about food bought would run out, food bought didn’t last, and their capacity to afford balanced meals in the past 12 months (0 = never true, 1 = sometimes true or often true). A food insecurity dummy (1 = food-insecure, 0 = food-secure) was created with 1 referring to households that experience at least one of the three difficulties, and 0 being those who never experience any of the three aspects of difficulties.

Dietary intake was measured by primary caregivers’ reports of children’s weekly intake of different food in the previous month. Two dummy indicators were created in the present study: **low vegetables and high sugar-sweetened beverages (SSBs**, 1 = yes, 0 = no), and **high carbohydrates** (1 = yes, 0 = no). Low vegetables and high SSBs was coded as “1” if children have green, leafy vegetables and fruits less than 5 times and take SSBs more than 3 times in a week, and “0” otherwise. High carbohydrates was coded as “1” if children consume all of the following food in a week: instant noodles; sweetened and salty snacks; fast food; and oily food. If children do not consume all of them, it was coded as “0”.

**Family economic stress** was measured by primary caregivers’ reports of whether the family has enough money to cover the expense incurred at the end of the month (1 = can’t make ends meet, 0 = can make ends meet).

**The primary caregiver’s depressive affect** was assessed using the Kessler Psychological Distress Scale [60]. The primary caregiver answered six questions about the frequency of feeling nervous, hopeless, restless, fidgety, overworked, sad, or worthless in the last month on a 5-point Likert Scale (1 = all of the time, 5 = none of the time). These items were reverse coded and averaged to generate the depressive affect index with the higher the score, the higher the depressive affect (Cronbach’s α was 0.84).

**Parenting style—**warm parenting and punitive parenting—was measured by primary caregivers’ reports of the interaction between themselves and the child. Warm parenting was captured by six items including the frequency the primary caregiver hugged or showed physical affection to the child in the past month (1 = not in the past month, 5 = everyday). Punitive parenting behavior was captured by five items such as the frequency of spanking the child, and taking away TV or other privileges (1 = not in the past month, 5 = everyday). Items of each subset were averaged to form a warm parenting index (Cronbach’s α was 0.89) and a punitive parenting index (Cronbach’s α was 0.64).

We include other covariates that have been shown in the literature to affect children’s behavior as control variable in the model. The child-level controls include children’s age, gender, ethnicity (ref. Chinese), low birth weight (0 = No, 1 = Yes), and children’s health status measured by whether the child has any chronic conditions (0 = No, 1 = Yes), and the number of siblings. It has been observed that households facing food insecurity often have a lower family income, restricted parental education, and an elevated rate of unemployment. Consequently, we have included certain family-level variables in our model. They are the highest education among parents (1 = secondary and below, 2 = post-secondary, 3 = university and above), the primary caregiver’s employment status (0 = employed, 1 = not employed), and family income quartile (1 = lowest quartile, 4 = highest quartile). The family structure which indicates whether the child was living with a single parent or not living with any parent (1 = Yes, 0 = No) was also controlled in the model.

### Analytic strategies

This study employed two main methods to examine the impact of food insecurity on children’s behavior problems: the propensity score matching analysis with wave 1 SG-LEADS data, and the fixed-effects model with two waves of data. The robustness of the result was tested by a set of lagged-variable models. The mediating mechanism of food insecurity on behavior problems was examined in the fixed-effects model. The analyses were conducted with STATA 17.

Propensity score matching method was used to control for systematic differences among children with different food-insecure statuses on selected covariates. Each child’s probability of experiencing food insecurity—propensity score—was estimated using generalized boosted modeling. Next, children with similar propensity scores yet different food-insecure statuses were matched to create samples that were balanced on the selected covariates. Optimal matching was adopted as the common support region across the treatment group (i.e., food-insecure children) and control group (i.e., food-secure children) is too narrow to permit the nearest neighbor matching. Three different types of matching were conducted: optimal full matching; optimal variable matching and optimal pair matching. The matching procedures were conducted with *optmatch* on R [61]. A covariate imbalance check after matching was conducted with a STATA program named imbalance [62]. Two different methods were employed for post-matching analysis to estimate the impact of food insecurity on children’s behavior problems: the Hodges-Lehmann aligned rank test for optimal full matching, and the difference-score regression for optimal pair matching.

Furthermore, a set of fixed-effects models was applied with two waves of data. The fixed-effects model accounts reasonably for unobservable individual-level characteristics and time-invariant factors. Therefore, it eliminates the potential omitted variable bias and provides an accurate estimation of the association between food insecurity and children’s behavior problems. To better understand the impact of food insecurity on children’s behavior problems, the mediating effect of the primary caregiver’s depressive affect and parenting, and children’s dietary intake was evaluated in the fixed-effects models. In the current analytical sample, around 30% of children were from the same household. The clustered standard error was applied to account for the unobserved household characteristics among children from the same household.

A set of lagged-variable models was used to test the robustness of the link between food insecurity and behavior problems. First, we used wave 1 food insecurity status to predict children’s behavior problems in wave 2. If the coefficient of wave 1 food insecurity is statistically significant, it suggests that the negative impact of food insecurity on children’s behavior problems could last for at least two years. Additionally, it rules out the possibility of reverse causality. Furthermore, we used wave 2 food insecurity to predict wave 2 behavior problems with wave 1 behavior problems being a control variable. By including the lagged dependent variable, we controlled for omitted or unobserved variables that may contribute to both food insecurity and children’s behavior problems.

Overall, the percentage of missing data is less than 1% for most of the variables in the current study, except for wave 1 behavior problems (3%). Thus, the list-wise deletion was adopted in the analysis.

## Results

Table 1 shows a comparison between children in food-secure households and food-insecure households on selected variables. At baseline, 10% of children experienced household-level food insecurity, and this percentage almost doubled in wave 2 (18%) during COVID-19 pandemic. In both waves, children in food-insecure households exhibited significantly higher externalizing and internalizing behavior problems than their food-secure peers. For dietary intake, food-insecure children were around 2 times more likely in both waves to have diets that are low in vegetables and high in SSBs. They consumed 2.7 times (W1) and 1.7 times (W2) more carbohydrates than the food-secure children. These food-insecure families were more than 10 times more likely to struggle financially, lacking sufficient money to make ends meet at the end of a month. The primary caregivers in these food-insecure households consistently exhibited higher depressive affect across both waves. Additionally, they used less warm parenting than their food-secure peers at baseline.

**Table 1:** Comparison between children in food-secure and food-insecure households (weighted).

	Wave 1		Wave 2	
	Food-secure	Food-insecure	Food-secure	Food-insecure
Weighted % (N)	90(2,190)	10(411)	82(1,978)	18(623)
**Dependent variables**	Proportion / Mean (SD)			
Externalizing BPI	1.39 (0.34)	1.59(0.35)*	1.48(0.34)	1.58(0.36)*
Internalizing BPI	1.13 (0.23)	1.28(0.32)*	1.21(0.24)	1.34(0.31)*
**Mediators**				
Low vegetables high SSBs	11	27*	12	22*
High carbohydrates	19	52*	35	61*
Can’t make ends meet	3	40 *	2	22*
PCG depressive affect	1.44(0.56)	2.11(0.76)*	1.85(0.66)	2.28(0.78)*
Punitive parenting	1.72(0.53)	1.76(0.58)	4.53(0.60)	4.51(0.60)
Warm parenting	4.84(0.37)	4.72(0.50)*	1.85(0.67)	1.91(0.73)
**Demographic controls**				
Child’s age	4.50(1.13)	4.47(1.15)	6.55(1.21)	6.53(1.22)
Boy	52	52	52	52
Child’s race				
Chinese	73	43*	73	43*
Malay	11	42*	11	42*
Indian	11	14	11	14
Others	6	2*	6	2*
Low birthweight	9	11	9	11
Chronic condition	7	12*	16	17
No. of siblings	1.23(0.89)	1.97(1.61)*	1.31(0.87)	1.82(1.41)*
Living with a single parent or without parent	4	10*	5	11*
Highest parent’ education				
Secondary and below	12	38*	10	33*
Post-secondary	27	43*	25	43*
University and above	61	19*	65	24*
PCG not employed	27	48*	23	43*
Income quartiles				
IncomeQ4 (highest)	26	3*	29	5*
IncomeQ3	26	9*	28	12*
IncomeQ2	27	18*	22	42*
Income Q1 (lowest)	22	71*	21	42*

*Note*. PCG refers to the primary caregiver.

* indicates the difference between children in food-insecure households and food-secure households is statistically significant (*p*<0.05).

The demographic profile varied significantly across these two types of households. A disproportionally higher share of children in Malay families were food-insecure than in other ethnic groups. On average, children in food-insecure families had more siblings, and a higher percentage of them were living with a single parent, or in some cases, without any parent. Their parents tended to have attained a secondary or below education, and their primary caregiver tended not to be in the labor force, and have a household income in the lowest quartile.

We conducted a propensity score matching analysis using the wave 1 dataset. The optimal full matching was adopted as it showed the lowest total distance without loss of cases and the highest bias reduction on the covariates. Results of both Ordinary Least Square (OLS) regression and post-matching analysis on the relationship between household food insecurity and children’s behavior are summarized in Table 2 (complete OLS regression results can be found in the online supplementary material S1 Table). In OLS regression, food insecurity manifested a statistically significant impact on children’s behavior problems. The coefficient was 0.20 for externalizing behavior problems, and 0.13 for internalizing behavior problems. These correlations persisted in the post-matching analysis after accounting for the selection bias introduced by the covariates. The average treatment effect of food insecurity on children’s behavior ranges from 0.11 to 0.17 for externalizing behavior problems, and 0.04 to 0.10 for internalizing behavior problems.

**Table 2:** Comparison of the estimated average treatment effect of household food insecurity on children’s externalizing and internalizing behavior across different models.

Models	Estimated average treatment effect (ATT)
**Externalizing BPI**	
OLS regression	0.20 ***
Optimal full matching with Hodges-Lehmann aligned rank test	0.17 ***
Regressing difference-score of outcome on difference scores of covariates after pair matching	0.11 ***
**Internalizing BPI**	
OLS regression	0.13 ***
Optimal full matching with Hodges-Lehmann aligned rank test	0.04 ***
Regressing difference-score of outcome on difference scores of covariates after pair matching	0.10 ***

*Note*. Covariates of the OLS model include children’s age, gender, ethnicity, children’s low birthweight, chronic conditions, number of siblings, whether the child lives with a single parent or not living with a parent, the parent’s education and employment status, family income quartile. OLS models were weighted.

Hodges-Lehmann aligned rank test is a bivariate and one-tailed test.

Covariates of different-score regression after pair matching include the child’s age, gender, ethnicity, family income quartile, primary caregiver’s depressive affect. These variables were selected as they were the main factors that affect the food insecurity propensity score.

* p<0.05, ** p<0.01

*** p<0.001.

To further account for unobservable individual-level characteristics and time-invariant factors, fixed-effect models were constructed with two waves of data. Results of fixed-effects models (Table 3) supported the adverse impact of food insecurity on children’s behavior problems (the complete model is available in the online supplementary material S2 Table). As seen, food insecurity positively led to children’s externalizing behavior problems with a coefficient of 0.11 (model 1), and internalizing behavior problems with a coefficient of 0.10 (model 5). The effect size is close to the results of regression on difference scores after pair matching. Our findings, obtained through various methods, consistently indicate that household food insecurity exacerbated young children’s externalizing and internalizing behavior problems.

**Table 3:** Fixed-effects models on children’s behavior problems (weighted).

	Externalizing BPI				Internalizing BPI			
	Model 1	Model 2	Model 3	Model 4	Model 5	Model 6	Model 7	Model 8
Food insecurity	0.110***	0.102***	0.072***	0.068***	0.103***	0.098***	0.073***	0.070***
	(0.0208)	(0.0206)	(0.0200)	(0.0200)	(0.0170)	(0.0169)	(0.0169)	(0.0169)
Low veggies high SSBs		0.095***		0.067***		0.039***		0.023
		(0.0186)		(0.0181)		(0.0148)		(0.0144)
High carbohydrates		0.028*		0.020		0.030***		0.024**
		(0.0149)		(0.0141)		(0.0116)		(0.0109)
Can’t make ends meet			0.045*	0.044*			0.028	0.027
			(0.0259)	(0.0259)			(0.0220)	(0.0221)
PCG’s depressive affect			0.060***	0.058***			0.055***	0.054***
			(0.0105)	(0.0105)			(0.00858)	(0.00858)
Warm parenting			-0.060***	-0.059***			-0.042***	-0.042***
			(0.0126)	(0.0125)			(0.0107)	(0.0107)
Punitive parenting			0.137***	0.133***			0.062***	0.061***
			(0.0118)	(0.0117)			(0.00881)	(0.00885)
Wave (1 = wave 2)	0.108***	0.098**	0.029	0.024	0.130***	0.129***	0.069**	0.062**
	(0.0397)	(0.0395)	(0.0378)	(0.0376)	(0.0274)	(0.0273)	(0.0268)	(0.0269)
Controls	Yes	Yes	Yes	Yes	Yes	Yes	Yes	Yes
Number of children	2,601	2,601	2,601	2,601	2,601	2,601	2,601	2,601
R-squared	0.064	0.075	0.165	0.171	0.097	0.100	0.159	0.160

Covariates of fixed-effects models include changes in the child’s age, number of chronic conditions, number of siblings, whether the child lives with a single parent or not living with a parent, parent’s education and employment status, family income quartile, and wave indicator.

Robust standard errors in parentheses.

*** p<0.01

** p<0.05

* p<0.1.

The mediating effect of children’s dietary intake and family stress was shown in models 2 to 4 and models 6 to 8 of Table 3. When controlling for dietary intake in the model, the coefficient of food insecurity declined slightly by 7% for externalizing behavior problems (model 2) and 5% for internalizing behavior problems (model 6). Consistent with the previous literature, we found a notable correlation between children’s dietary intake—low vegetables and high SSBs, and high carbohydrates—and their externalizing and internalizing behavior problems. The results suggest that children’s food intake partially mediated the impact of food insecurity on children’s behavior problems.

In models 3 and 7, when family economic stress and parenting were adjusted for in the model, the coefficient of food insecurity decreased by around 30% for both externalizing and internalizing behavior problems. The coefficients of primary caregivers’ emotional distress, and warm and punitive parenting were statistically significant. These results suggest that family stress explained roughly one-third of the effect of food insecurity on children’s behavior problems. This lends support to the family stress hypothesis that food insecurity is an environmental stress potentially impairing the primary caregivers’ psychological well-being. Such family-level stress influences caregivers’ parenting styles and, ultimately, children’s behavior problems.

In model 4 and model 8, when constructs of family stress and nutrition pathways were included simultaneously, most mediators of each pathway retained their statistical significance. The findings revealed that the impact of food insecurity on children’s externalizing and internalizing behavior problems was mediated by both the family stress pathway and nutrition pathway. It is noteworthy that the family stress pathway had a larger explanatory power than the nutrition pathway. This result has also been confirmed in mediation analysis.

### Robustness analysis

The robustness of the results was tested through lagged-variable models and presented in Table 4 (the complete model can be found in S3 Table in the online Appendix). Model 1 indicates that children living in food-insecure households at baseline were significantly more likely to exhibit externalizing behavior problems two years later during wave 2 (b = 0.07, p<0.05), after controlling for covariates. In model 2, it is found that wave 2 food insecurity significantly and positively influenced (b = 0.07, *p*<0.05) children’s externalizing behavior problems concurrently after controlling for externalizing behavior problems at baseline. Similarly, wave 1 food insecurity showed a sustained impact on children’s internalizing behavior problems in wave 2 (b = 0.07, *p*<0.05, model 3). Living in a food-insecure household in wave 2 also positively contributed to wave 2 internalizing behavior problems concurrently net of wave 1 internalizing behavior problems (b = 0.11, *p*<0.05, model 4). The results of lagged-variable models confirmed that food insecurity not only had a concurrent effect on children’s behavior problems, but also a sustained impact observed two years later. Findings here also ruled out the potential reverse causality that children’s behavior problems increase their food insecurity.

**Table 4:** Lagged models of food insecurity on wave 2 behavior problems (weighted).

	W2 Externalizing BPI		W2 Internalizing BPI	
	Model 1	Model 2	Model 3	Model 4
**Independent variables**				
W1 Food insecurity	0.0681**		0.0715***	
	(0.0335)		(0.0243)	
W2 Food insecurity		0.0728***		0.113***
		(0.0227)		(0.0188)
**Lagged dependent variables**				
W1 Externalizing BPI		0.263***		
		(0.0301)		
W1 Internalizing BPI				0.204***
				(0.0493)
Controls	Yes	Yes	Yes	Yes
Observations	2,558	2,558	2,558	2,558
R-squared	0.062	0.132	0.078	0.133

Controls include children’s age, gender, ethnicity, low birthweight, chronic conditions, number of siblings, whether the child lives with a single parent or not living with a parent, the parent’s education, employment status, family income quartile.

Robust standard errors in parentheses.

*** p<0.01

** p<0.05

* p<0.1.

## Discussion

This study investigated the causal association between household food insecurity and young Singaporeans’ behavior problems with two waves of data from a nationally representative longitudinal dataset—SG-LEADS. Corroborating previous research, the study revealed that household food insecurity moderately increased young children’s externalizing and internalizing behavior problems both concurrently and two years later when holding other factors constant. We further elucidated two mediating pathways through which food hardship influenced children’s behavior—the nutrition pathway and the family stress pathway. Align with previous literature [27,29,39], the effect of food insecurity on children’s behavior could be attributed to their dietary intake of high carbohydrates and refined sugar and low fruits and vegetables, which affected children’s behavior problems. We also found that parental depressive affect and parenting behavior largely explained the link between household food insecurity and children’s behavior problems. Food insecurity was positively correlated with parental psychological stresses, which disrupted family relationships and effective parenting by reducing parental warmth and increasing harsh disciplines. These parenting styles were associated with more externalizing and internalizing behavior problems among children.

The COVID-19 pandemic introduced unprecedented economic challenges and exacerbated food insecurity, hitting disadvantaged households particularly hard. Although food assistance can be obtained in Singapore from charities, it is found that only a small proportion of food insecure households have sought help probably due to social embarrassment and unawareness of food support [51]. This social embarrassment adds another layer of stress to these food-insecure households. It is important to note that the social determinants (family stress pathway) had a larger explanatory power than the biological factors (nutrition pathway) in affecting children’s behavior problems. Therefore, it’s imperative that interventions targeting food insecurity not only address the immediate need for nutrition but also tackle the psychosocial stress of food-insecure families.

This study contributes to the literature in several ways. Firstly, it provides evidence for the impact of household food insecurity on young children’s behavior problems during the COVID-19 pandemic with robust statistical methods. The propensity score matching analysis addresses the endogeneity and selection bias of the sample, and the fixed-effects model based on two waves of data controls for the effect of individual-level time-invariant factors. Furthermore, the robustness analysis with lagged-variable models rules out the possibility of reverse causality. Secondly, the present study examines two distinct mediating pathways simultaneously and compare their relative importance—the family stress pathway and the nutrition pathway. The nutrition pathway has not been explored in previous studies due to data limitations, which adds novelty to our findings hereby. Thirdly, the study sheds light on the impact of food insecurity during the pandemic on young children’s development. This analysis is of utmost importance due to the significant increase in food insecurity caused by the COVID-19 pandemic, with varying impacts observed across different countries. The study contributes to our knowledge of how the pandemic has exacerbated food insecurity and its specific implications for children’s well-being. Lastly, by leveraging a recent nationally representative dataset from Singapore, this study expands upon findings typically reported in Western contexts. The results echo those from Western countries, enhancing the generalizability of the association between food insecurity and young children’s behavior problems and the two mediating mechanisms.

We note some limitations of this study. First, although the study uses robust statistical methods with a longitudinal dataset to strengthen the causal link, we cannot claim causality without doubt as omitted controls or unobserved time-variant factors that contribute to both food insecurity and children’s behavior problems may still present [25,63]. However, given the relatively short interval between the two waves of the survey—two years—omitted time-variant factors were less likely to change in a way that substantially affects the estimates. Secondly, the study is based on two waves of data, which does not account for the long-term effect of food insecurity on children’s behavior. Thus, further studies incorporating multiple waves of data are imperative for a more comprehensive understanding of this critical issue.

## Conclusion

The present study expands the previous literature by evaluating how household food insecurity during the COVID-19 pandemic affects young children’s behavior problems, and jointly tests two distinct mediating mechanisms involved in a non-Western context. The analyses provide compelling empirical evidence indicating that household food insecurity exerts an adverse impact on young children’s externalizing and internalizing behavior problems through multifaceted pathways, including the nutrition pathway and family stress pathway. This underscores the importance of recognizing that food insecurity has negative intergenerational consequences on children’s socioemotional development. Interventions are needed to identify vulnerable families, particularly those with young children, and help them to reduce food insecurity, alleviate family stress and improve children’s nutrition to facilitate children’s healthy social-emotional development.

## Supporting information

S1 Dataset Minimal dataset. (XLSX)

S1 Table Average treatment effect of household food insecurity on children’s externalizing and internalizing behavior in OLS model (weighted). (DOC)

S2 Table Fixed effects models on children’s behavior problems (weighted). (DOC)

S3 Table Lagged models of food insecurity on wave 2 behavior problems (weighted). (DOC)
