# Supplementary material for: How food insecurity affects children’s behavior problems in early childhood: The nutrition and family stress pathways
Source: PLoS One. 2024 Jan 3;19(1):e0294109. doi: 10.1371/journal.pone.0294109 (PMC10763944; doi:10.1371/journal.pone.0294109)
Supplement: S1 Table — (DOC) [file pone.0294109.s002.doc]

**S1 Table. Average treatment effect of household food insecurity on children’s externalizing and internalizing behavior in OLS model (weighted).**

|  | Model 1 | Model 2 |
| --- | --- | --- |
| VARIABLES | Externalizing BPI | Internalizing BPI |
|  |  |  |
| W1 age | -0.0161** | 0.0104** |
|  | (0.00671) | (0.00442) |
| Boy | 0.0345* | 0.000625 |
|  | (0.0184) | (0.0153) |
| Ethnicity (ref. Chinese) |  |  |
| Malay | -0.0988*** | -0.0525** |
|  | (0.0251) | (0.0205) |
| Indian | -0.00977 | -0.000459 |
|  | (0.0257) | (0.0194) |
| Others | 0.0133 | -0.0725*** |
|  | (0.0473) | (0.0207) |
| W1 chronic conditions | 0.110*** | 0.107*** |
|  | (0.0276) | (0.0289) |
| Low birthweight | 0.00900 | -0.00917 |
|  | (0.0282) | (0.0185) |
| W1 single no parent | -0.00367 | 0.00288 |
|  | (0.0108) | (0.00919) |
| W1 PCG not working | 0.00108 | -0.0362* |
|  | (0.0409) | (0.0207) |
| Low birthweight | -0.0253 | -0.00565 |
|  | (0.0197) | (0.0144) |
| W1 parent’s education (Ref. University and above) | | |
| Post-Secondary | 0.0476** | 0.0245* |
|  | (0.0212) | (0.0141) |
| Secondary and below | -0.00810 | -0.00549 |
|  | (0.0287) | (0.0197) |
| W1 Income quartile (Ref. Q4 highest) | | |
| incomeQ3 | 0.00854 | 0.00771 |
|  | (0.0298) | (0.0246) |
| incomeQ2 | 0.0151 | 0.0119 |
|  | (0.0300) | (0.0240) |
| incomeQ1 lowest | 0.0684** | 0.0586** |
|  | (0.0315) | (0.0244) |
| W1 food insecurity | 0.200*** | 0.133*** |
|  | (0.0280) | (0.0233) |
| Constant | 1.424*** | 1.064*** |
|  | (0.0377) | (0.0261) |
|  |  |  |
| Observations | 2,559 | 2,559 |
| R-squared | 0.066 | 0.078 |

Robust standard errors in parentheses

*** p<0.01, ** p<0.05, * p<0.1
