# Supplementary material for: How food insecurity affects children’s behavior problems in early childhood: The nutrition and family stress pathways
Source: PLoS One. 2024 Jan 3;19(1):e0294109. doi: 10.1371/journal.pone.0294109 (PMC10763944; doi:10.1371/journal.pone.0294109)
Supplement: S2 Table — (DOC) [file pone.0294109.s003.doc]

**S2 Table. Fixed effects models on children’s behavior problems** **(weighted).**

| VARIABLES | Externalizing BPI | | | | Internalizing BPI | | | |
| --- | --- | --- | --- | --- | --- | --- | --- | --- |
|  | Model 1 | Model 2 | Model 3 | Model 4 | Model 5 | Model 6 | Model 7 | Model 8 |
| Food insecurity | 0.110*** | 0.102*** | 0.0723*** | 0.0681*** | 0.103*** | 0.0984*** | 0.0727*** | 0.0702*** |
|  | (0.0208) | (0.0206) | (0.0200) | (0.0200) | (0.0170) | (0.0169) | (0.0169) | (0.0169) |
| Low veggies high SSB |  | 0.0949*** |  | 0.0670*** |  | 0.0388*** |  | 0.0229 |
|  |  | (0.0186) |  | (0.0181) |  | (0.0148) |  | (0.0144) |
| High carbohydrate |  | 0.0279* |  | 0.0199 |  | 0.0301*** |  | 0.0237** |
|  |  | (0.0149) |  | (0.0141) |  | (0.0116) |  | (0.0109) |
| Can’t make ends meet |  |  | 0.0454* | 0.0440* |  |  | 0.0276 | 0.0265 |
|  |  |  | (0.0259) | (0.0259) |  |  | (0.0220) | (0.0221) |
| PCG depressive affect |  |  | 0.0602*** | 0.0577*** |  |  | 0.0554*** | 0.0539*** |
|  |  |  | (0.0105) | (0.0105) |  |  | (0.00858) | (0.00858) |
| Warm parenting |  |  | -0.0595*** | -0.0590*** |  |  | -0.0421*** | -0.0420*** |
|  |  |  | (0.0126) | (0.0125) |  |  | (0.0107) | (0.0107) |
| Punitive parenting |  |  | 0.137*** | 0.133*** |  |  | 0.0620*** | 0.0607*** |
|  |  |  | (0.0118) | (0.0117) |  |  | (0.00881) | (0.00885) |
|  |  |  |  |  |  |  |  |  |
| Age | -0.0130 | -0.0109 | -0.00123 | -0.000177 | -0.0241* | -0.0224* | -0.0140 | -0.0129 |
|  | (0.0190) | (0.0187) | (0.0178) | (0.0176) | (0.0132) | (0.0131) | (0.0125) | (0.0125) |
| chronic conditions | 0.0303* | 0.0302* | 0.0224 | 0.0226 | 0.00345 | 0.00421 | -0.00261 | -0.00178 |
|  | (0.0162) | (0.0161) | (0.0164) | (0.0163) | (0.0133) | (0.0133) | (0.0132) | (0.0132) |
| Number of Siblings | -0.00196 | -0.00738 | -0.0232 | -0.0265 | 0.0109 | 0.00811 | -0.000175 | -0.00177 |
|  | (0.0256) | (0.0252) | (0.0239) | (0.0237) | (0.0166) | (0.0165) | (0.0156) | (0.0156) |
| Not living with 2 parents | -0.0695 | -0.0642 | -0.0442 | -0.0410 | -0.0217 | -0.0185 | -0.00879 | -0.00669 |
|  | (0.0501) | (0.0499) | (0.0423) | (0.0425) | (0.0420) | (0.0419) | (0.0380) | (0.0380) |
| parent’s education (Ref. University and above) | | | | | | | | |
| Post-Secondary | 0.145** | 0.149** | 0.0892 | 0.0944 | 0.102*** | 0.103*** | 0.0679* | 0.0693* |
|  | (0.0727) | (0.0729) | (0.0647) | (0.0648) | (0.0382) | (0.0382) | (0.0386) | (0.0385) |
| Secondary and below | 0.241** | 0.230** | 0.200** | 0.193** | 0.174*** | 0.168*** | 0.145** | 0.142** |
|  | (0.0984) | (0.0989) | (0.0928) | (0.0932) | (0.0608) | (0.0616) | (0.0618) | (0.0621) |
| Income quartile (Ref. Q4 highest) | | | | | | | | |
| incomeQ3 | -0.00694 | -0.00741 | -0.0280 | -0.0279 | -0.0269 | -0.0271 | -0.0397** | -0.0396** |
|  | (0.0238) | (0.0236) | (0.0218) | (0.0218) | (0.0166) | (0.0166) | (0.0157) | (0.0156) |
| incomeQ2 | 0.00405 | 0.00402 | -0.00192 | -0.00183 | -0.0256 | -0.0253 | -0.0273* | -0.0270* |
|  | (0.0233) | (0.0231) | (0.0214) | (0.0213) | (0.0156) | (0.0155) | (0.0147) | (0.0146) |
| incomeQ1 lowest | -0.00547 | -0.00534 | -0.00944 | -0.00926 | -0.00858 | -0.00862 | -0.0102 | -0.0102 |
|  | (0.0234) | (0.0232) | (0.0218) | (0.0217) | (0.0155) | (0.0154) | (0.0146) | (0.0145) |
| PCG not working | -0.0128 | -0.0191 | -0.0199 | -0.0243 | -0.0174 | -0.0200 | -0.0210 | -0.0225 |
|  | (0.0305) | (0.0309) | (0.0290) | (0.0294) | (0.0225) | (0.0229) | (0.0215) | (0.0217) |
| Wave | 0.108*** | 0.0983** | 0.0288 | 0.0241 | 0.129*** | 0.120*** | 0.0681** | 0.0623** |
|  | (0.0397) | (0.0395) | (0.0378) | (0.0376) | (0.0274) | (0.0275) | (0.0268) | (0.0269) |
| Constant | 1.376*** | 1.358*** | 1.345*** | 1.339*** | 1.197*** | 1.182*** | 1.199*** | 1.192*** |
|  | (0.104) | (0.103) | (0.114) | (0.114) | (0.0692) | (0.0691) | (0.0843) | (0.0842) |
|  |  |  |  |  |  |  |  |  |
| Observations | 5,159 | 5,159 | 5,157 | 5,157 | 5,159 | 5,159 | 5,157 | 5,157 |
| R-squared | 0.066 | 0.079 | 0.167 | 0.173 | 0.097 | 0.103 | 0.159 | 0.162 |
| Number of children | 2,601 | 2,601 | 2,601 | 2,601 | 2,601 | 2,601 | 2,601 | 2,601 |

Robust standard errors in parentheses

*** p<0.01, ** p<0.05, * p<0.1
