# Supplementary material for: How food insecurity affects children’s behavior problems in early childhood: The nutrition and family stress pathways
Source: PLoS One. 2024 Jan 3;19(1):e0294109. doi: 10.1371/journal.pone.0294109 (PMC10763944; doi:10.1371/journal.pone.0294109)
Supplement: S3 Table — (DOC) [file pone.0294109.s004.doc]

**S3 Table. Lagged models of food insecurity on wave 2 behavior problems (weighted).**

|  | Model 1 | Model 2 | Model 3 | Model 4 |
| --- | --- | --- | --- | --- |
| VARIABLES | W2  Externalizing BPI | W2  Externalizing BPI | W2  Internalizing BPI | W2  Internalizing BPI |
|  |  |  |  |  |
| W2 age | -0.0133** | -0.00921 | 0.0152*** | 0.0134*** |
|  | (0.00622) | (0.00598) | (0.00448) | (0.00437) |
| Boy | 0.0574*** | 0.0472*** | -0.000776 | -0.00301 |
|  | (0.0159) | (0.0157) | (0.0110) | (0.0110) |
| Ethnicity (ref. Chinese) |  |  |  |  |
| Malay | -0.104*** | -0.0891*** | -0.0379** | -0.0414** |
|  | (0.0238) | (0.0217) | (0.0174) | (0.0165) |
| Indian | -0.118*** | -0.121*** | -0.0547*** | -0.0622*** |
|  | (0.0265) | (0.0254) | (0.0188) | (0.0180) |
| Others | -0.164*** | -0.165*** | -0.114*** | -0.0914*** |
|  | (0.0538) | (0.0523) | (0.0304) | (0.0276) |
| W1 chronic conditions | 0.113*** | 0.0828*** | 0.116*** | 0.0930*** |
|  | (0.0225) | (0.0199) | (0.0184) | (0.0180) |
| Low birthweight | 0.0404 | 0.0357 | 0.0264 | 0.0250 |
|  | (0.0287) | (0.0266) | (0.0202) | (0.0190) |
| W1 single no parent | 0.103** | 0.0932** | 0.0461 | 0.0394 |
|  | (0.0424) | (0.0414) | (0.0315) | (0.0293) |
| W1 PCG not working | -0.00744 | -0.00170 | -0.0122 | -0.0123 |
|  | (0.0194) | (0.0183) | (0.0140) | (0.0138) |
| W1 parent’s education (Ref. University and above) |  |  |  |  |
| Post-Secondary | 0.0502** | 0.0322 | 0.0256 | 0.0117 |
|  | (0.0228) | (0.0222) | (0.0158) | (0.0151) |
| Secondary and below | 0.0205 | 0.0110 | 0.00848 | -0.00672 |
|  | (0.0293) | (0.0283) | (0.0209) | (0.0208) |
| W1 Income quartile (Ref. Q4 highest) |  |  |  |  |
| incomeQ3 | 0.0228 | 0.0197 | 0.0487*** | 0.0470*** |
|  | (0.0255) | (0.0251) | (0.0161) | (0.0165) |
| incomeQ2 | 0.0541** | 0.0485* | 0.0661*** | 0.0625*** |
|  | (0.0259) | (0.0256) | (0.0168) | (0.0172) |
| incomeQ1 lowest | 0.0523* | 0.0217 | 0.0799*** | 0.0532** |
|  | (0.0298) | (0.0287) | (0.0214) | (0.0213) |
| W1 food insecurity | 0.0681** |  | 0.0715*** |  |
|  | (0.0335) |  | (0.0243) |  |
| W2 food insecurity |  | 0.0728*** |  | 0.113*** |
|  |  | (0.0227) |  | (0.0188) |
| W1 externalizing BPI |  | 0.263*** |  |  |
|  |  | (0.0301) |  |  |
| W1 internalizing BPI |  |  |  | 0.204*** |
|  |  |  |  | (0.0493) |
| Constant | 1.514*** | 1.133*** | 1.067*** | 0.851*** |
|  | (0.0441) | (0.0607) | (0.0308) | (0.0554) |
|  |  |  |  |  |
| Observations | 2,558 | 2,558 | 2,558 | 2,558 |
| R-squared | 0.062 | 0.132 | 0.078 | 0.133 |

Robust standard errors in parentheses

*** p<0.01, ** p<0.05, * p<0.1
